# Decoding the growth–defense dialectic: TOR signaling and developmental genetics in maize

**DOI:** 10.1093/jxb/eraf358

**Published:** 2025-08-11

**Authors:** Michael Busche, Sannidhi Menon, Jacob O Brunkard

**Affiliations:** Laboratory of Genetics, University of Wisconsin—Madison, 425 Henry Mall, Madison, WI 53706, USA; Laboratory of Genetics, University of Wisconsin—Madison, 425 Henry Mall, Madison, WI 53706, USA; Laboratory of Genetics, University of Wisconsin—Madison, 425 Henry Mall, Madison, WI 53706, USA; Aix-Marseille Université, France

**Keywords:** Development, growth–defense, ligule, maize genetics, stress response, TARGET OF RAPAMYCIN, *Zea mays*

## Abstract

Plants face diverse abiotic and biotic stresses, including drought, heat, salinity, herbivory, pathogens, and competition. To mitigate the fitness costs of these threats, they have evolved immediate compensatory mechanisms and immune responses, such as phytohormone signaling, secondary metabolite production, and the hypersensitive response. However, activating these stress-response programs often comes at the expense of optimal growth. This shift in cellular energy and resource allocation underpins the classical ‘growth–defense trade-off’. Beyond short-term metabolic reprogramming, plants also engage developmental switches that alter broader growth patterns to compensate for or avoid stress. In this review, we explore how maize, a longstanding model for plant development, rewires growth in response to stress. We highlight key developmental genes that maintain homeostatic growth or trigger major morphological changes in coordination with stress signals. We also examine recent insights into how plants rebalance energy under stress, with a focus on the TOR-sensitive hormone networks. Finally, we discuss how maize-specific innovations in growth–stress integration could inform efforts to enhance resilience in other crops. These strategies are essential for developing more sustainable agriculture, where crops can endure transient stress without initiating permanent developmental shifts that reduce yield.

## Teaser

We review dynamic regulation of plant growth and development in response to stress through the TOR signaling network, with a focus on maize developmental biology and growth–defense trade-offs in agriculture and beyond.

## Introduction

After germination, plants continually generate new organs, including leaves, roots, and reproductive structures (Sluis and Hake, 2015). Their indeterminate body plan is critical to plant success, enabling dynamic adjustments of growth in response to current environmental conditions. For example, during post-embryonic organogenesis, plants fine-tune the scale and tempo of development in response to external cues. When conditions are favorable, plants grow robustly and proceed through development at a predictable pace and sequence. When conditions are unfavorable, plants can alter their development in several ways, for instance to avoid starvation when nutrients are limiting or to defend from pathogen attack, ultimately attempting to maintain metabolic homeostasis and successfully reproduce despite dynamically stressful conditions (Hey *et al*., 2010). In modern agriculture, however, even brief environmental stress can derail developmental programs in ways that have lasting effects on plant performance and yield.

These developmental alterations can include major changes to vegetative and reproductive growth and development, including the size, shape, and number of organs produced during a plant’s lifecycle, as well as the timing of their emergence. Some plants can even change their reproductive lifestyle in response to their environment: *Mimulus guttatus* can be perennial if it grows near the coast, but if it grows on mountains it switches to an annual life cycle because of drought stress during summers (Baker and Diggle, 2011). In crop species, these stress-induced developmental responses can mean changes in plant architecture, flowering time, fruit setting and filling, and, ultimately, yield (Kazan and Lyons, 2016; Ruan *et al*., 2017).

Changes in developmental strategy in response to stress are, in part, due to a shift from ‘normal’ physiology and metabolic pathways to those aimed at compensating for or avoiding a given stress. The switch between growth and a stress response is referred to as the growth–defense trade-off. The theory of a growth–defense dichotomy in plants originally emerged to describe an observed switch within metabolism and gene expression in response to herbivory (Feeny, 1976; Rhoades and Cates, 1976; van der Meijden *et al*., 1988). In instances of herbivory, some plants temporarily reorganize their metabolism to preferentially produce metabolites that reduce digestibility or are toxic to pests. This reorganization channels metabolic resources away from primary metabolic and physiological programs that support growth. At the same time, plants can actively down-regulate growth in response to pathogen stress to delay the formation of new tissue and protect themselves from damage or infection. In summary, these strategies largely place defense and growth in direct opposition (Guo *et al*., 2018; He *et al*., 2022; Monson *et al*., 2022). Although this switch was initially posited to describe the relationship between growth and defense from biotic stress specifically, it has been repeatedly shown to accurately describe the relationship between growth and response to stress more broadly.

In fact, there is often overlap in the strategies that plants use to address individual stresses. When primed by an initial stress, in a process known as cross-adaptation or ‘hardening’, plants will then show increased resistance to subsequent or seemingly unrelated stresses (Bowler and Fluhr, 2000; Mittler, 2006; Foyer *et al*., 2016). For example, abscisic acid (ABA) is implicated in the response to both heat and cold stress, as well as to drought and salinity stress. Up-regulation of ABA production and signaling in response to heat has been shown to increase drought tolerance (Sah *et al*., 2016).

Beyond immediate changes to metabolism and the transient adjustments that come with them (e.g. stomatal closure), long-term exposure to stress can substantially alter plant development. These multi-layered changes include premature senescence, early or delayed flowering, and impaired reproduction, among others. Although advantageous in the wild, prolonged delays in growth and major developmental changes are undesirable in an agricultural setting. Consequently, modern maize has been intensively bred to continue growing ‘normally’ under a wide range of adverse conditions. For instance, the shade avoidance syndrome has largely been bred out of maize to allow for extreme planting density (Ku *et al*., 2015; Jafari *et al*., 2024). Meanwhile, stay-green traits have been introgressed into modern hybrids to prevent senescence and extend the photosynthetic period to increase grain-filling (Zhang *et al*., 2019).

Efforts like these that prioritize growth have come at a cost, however. By intentionally eroding stress responses to favor growth, modern maize has become more susceptible to abiotic and biotic stresses when compared with ancestral or landrace varieties (Prasanna, 2012). This has led to a push to identify and introduce (or re-introduce) biotic and abiotic stress-tolerance traits from wild relatives. Traits from *Zea nicaraguensis*, *Z. luxurians*, *Z. mays* subsp. *huehuetenangensis*, and *Z. diploperennis* have been introduced into modern maize to increase resistance to stresses including flooding, drought, and pest damage (Mano *et al*., 2005, 2006; Shaibu *et al*., 2021). While many loci for stress tolerance have been identified (Sheoran *et al*., 2022), both the immediate and long-term strategies to accommodate stress are influenced by many genes. Furthermore, the genes that coordinate stress responses, as well as the secondary metabolites and phytohormones active in such responses, often interact with each other and/or are heavily influenced by the environment.

This complex crosstalk and environmental interaction make understanding the growth–-defense trade-off complicated, but it remains critically important for maize biologists. Climate change has already begun to impact food systems around the world, and the threats it poses to food security are only projected to increase (Malhi *et al*., 2021; Farooq *et al*., 2023). This danger is increasingly relevant to plant biology, and many scientists are working to understand how plants manage the complicated relationship between growth and response to stress (Eckardt *et al*., 2023; Sugumar *et al*., 2024).

Therefore, new strategies for incorporating traits to enhance maize stress resistance continue to be developed (Malenica *et al*., 2021). Improved gene editing and plant transformation technologies (e.g. Szarzanowicz *et al*., 2024) are likely to accelerate production of crop varieties with enhanced resistance to diverse stresses (Rai *et al*., 2023). However, to date only a handful of improved stress-tolerant crop varieties have been realized in the field, underscoring the genetic complexity of stress tolerance traits. For instance, transgenic maize engineered to reduce trehalose-6-phosphate (T6P) exhibits yield gains of 30–120% under drought in field trials—an unfortunately rare example of a genetic modification translating to robust field performance (Nuccio *et al*., 2015). Continued fundamental research is needed to prevent over-promising for outcomes and to identify high-impact editing targets (Bailey-Serres *et al*., 2019; Karavolias *et al*., 2021). Studying the genetic mechanisms underlying the patterning of crop development, along with the interactions of these genes with each other and the environment, enables us to more precisely design new crop varieties that maximize yield in the face of varied stresses.

In this review, we consider a few recent examples of work that explore the genetic regulation of the growth–defense balance. We not only cover how stress impacts growth and development, but also how changes in growth can enhance sensitivity to stress. We focus on the master growth regulator TARGET OF RAPAMYCIN (TOR) and consider genes in the TOR signaling network as potential targets for genetic engineering to rebalance crop homeostasis in the face of changing environments. We hope to provide perspective on the results of current research in plant growth–defense trade-offs, to comment on important next steps to understanding plant energy rebalancing, and to propose mechanisms by which the TOR signaling network might provide solutions to the problems facing modern agriculture.

## Plant energy management

Plant development is inherently dynamic, requiring continuous adjustment as plants generate new tissues and organs throughout their lifetimes. This ongoing organogenesis provides plants with a remarkable flexibility, enabling them to tailor their growth strategies to fluctuating environmental conditions. Central to this adaptability is the careful regulation of resource allocation: when nutrients are abundant and stress is low, plants invest heavily in growth, producing leaves, roots, and reproductive structures in predictable patterns. Conversely, when conditions become challenging, such as during nutrient scarcity or pathogen stress, plants strategically redistribute energy away from growth processes toward defense and survival pathways. This shift prioritizes immediate survival while also maintaining metabolic homeostasis, ultimately safeguarding reproductive success in an ever-changing environment (Heil, 2010; Albrecht and Argueso, 2017).

TARGET OF RAPAMYCIN (TOR) is a eukaryotic serine/threonine protein kinase that monitors internal nutrient status (i.e. amino acids, nucleotides, and energy availability), and when such nutrients are sufficient it stimulates translation, ribosome biogenesis, and cell division (Saxton and Sabatini, 2017; Scarpin *et al*., 2022). *TOR* is an essential gene and is necessary for progression through development: silencing or inhibiting it leads to complete arrest of growth (Xiong *et al*., 2013; Chatterjee *et al*., 2021, Preprint; Busche *et al*., 2021). TOR has been well-studied by biologists working in biomedically relevant systems due to its importance in human health and disease, but has only gained major attention from plant biologists in the last ∼20 years (Mahfouz *et al*., 2006; Deprost *et al*., 2007). It has been shown that in plants TOR does indeed sense nutrient abundance (including sugars, nucleotides, and ATP) and coordinates growth and development in response to their status (Xiong *et al*., 2013; Brunkard *et al*., 2020; Scarpin *et al*., 2020; Busche *et al*., 2021). For instance, when photosynthetically active radiation (PAR) becomes limiting, energy in the form of glucose and ATP becomes less available, likely preventing TOR activation and ultimately hampering growth (Amthor, 2010). Furthermore, TOR appears to be light sensitive: in response to changes in light, it modulates phytochrome and phytohormone signaling (Pfeiffer *et al*., 2016; Riegler *et al*., 2021; Berry and Argueso, 2022; Busche, 2023; Saile *et al*., 2023). Evidence suggests that TOR not only regulates growth in response to nutrient and energy status, but that it also acts as a critical node in stress and defense-signaling responses, dynamically adjusting growth in response to stress when conditions demand it (Reitz *et al*., 2015).

In addition to phytohormones, metabolic signals such as T6P directly influence TOR signaling and stress responses. T6P levels reflect sucrose status, and high levels can activate TOR and promote growth. Conversely, low levels relieve inhibition of SNF1-RELATED KINASE (SnRK1), leading to stress-response metabolic programs. In maize, altering T6P/SnRK1 activity shifts resource allocation between primary (growth) and secondary (defense) metabolism. Notably, boosting T6P turnover via transgenic T6P phosphatase increases maize yield under drought stress, highlighting the potential of sugar-signaling pathways in managing growth–defense trade-offs (Nuccio *et al*., 2015; Flavell, 2023)

## TOR–ABA antagonism

**Fig. 1.**
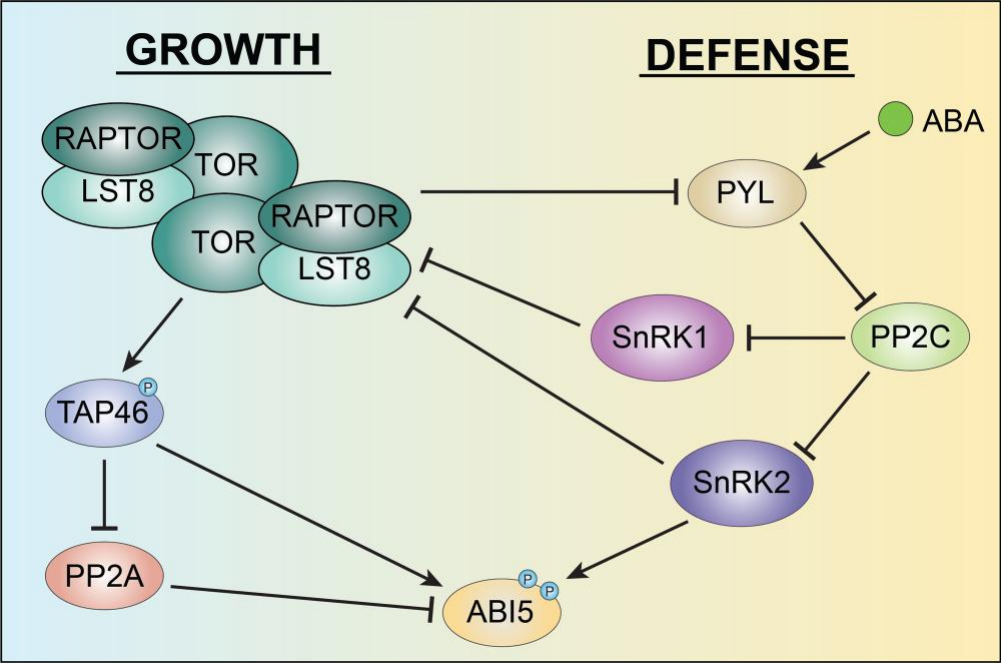
ABI5 coordinates growth–defense trade-offs by integrating signals from the TOR Complex 1 and ABA. A ‘tug-of-war’ between growth and defense signaling pathways converge at the ABA-sensitive transcription factor ABI5. Under stress, ABA binds to its receptor PYL, which then binds to and inhibits PP2C. This inhibition allows levels of phosphorylated SnRK2 to build up and activate ABA-sensitive transcription factors such as ABI5 (Cutler *et al*., 2010). Under growth-promoting conditions, TOR phosphorylates PYL, preventing it from de-repressing SnRK2. Both sides of the antagonism can function simultaneously, and they continually feedback to regulate the activity of the other. The canonical TOR effector TAP46 also protects ABI5 from dephosphorylation via PP2A, thereby stabilizing the ABA response. This mutual regulation between growth and defense ensures neither process overwhelms the other. ABA, abscisic acid; LST8, LETHAL WITH SEC THIRTEEN 8; PP2C, PROTEIN PHOSPHATASE 2C; RAPTOR, REGULATORY ASSOCIATED PROTEIN OF mTOR; SnRK1, SNF1-RELATED KINASE; TAP46, TYPE 2A PHOSPHATASE-ASSOCIATED PROTEIN OF 46 kDa; TOR, TARGET OF RAPAMYCIN.

Mechanistically, TOR acts through the TOR Complex 1 (TORC1), which in plants consists of the scaffold protein REGULATORY ASSOCIATED PROTEIN OF mTOR (RAPTOR), and LETHAL WITH SEC THIRTEEN 8 (LST8) (Fig. 1). TORC1 selects and directly phosphorylates key proteins to drive growth. One prominent target of TOR is the regulatory protein TYPE 2A PHOSPHATASE-ASSOCIATED PROTEIN OF 46 kDa (TAP46), a subunit of PROTEIN PHOSPHATASE 2A (PP2A). TAP46 is a conserved TOR effector across plants (Ahn *et al*., 2011), and its disruption in Arabidopsis impairs growth and leads to metabolic imbalance, suggesting a functional link to TOR signaling (Rahikainen *et al*., 2016). Through TAP46, active TOR likely maintains certain stress-response factors in an inactive state under favorable conditions.

Notably, TAP46 also intersects with the stress-sensitive phytohormone ABA. TAP46 binds the ABA-responsive transcription factor ABI5 and protects it from dephosphorylation (inactivation) by PP2A, thereby stabilizing ABA signaling outputs (Fig. 1) (Postiglione and Muday, 2023). Overexpression of TAP46 enhances ABA sensitivity in germination assays, whereas loss of TAP46 confers ABA insensitivity, indicating that it is a positive regulator of ABA-mediated stress responses (Hu *et al*., 2014). These findings suggest that TAP46 might sit at nexus of the TOR network and the ABA pathway, potentially allowing TOR to influence ABA-responsive gene expression via control of PP2A/ABI5 activity.

Accumulating evidence points to reciprocal regulation between growth-promoting TOR signals and stress-activated ABA signals. In well-watered, non-stress conditions, TOR activity tends to keep ABA responses repressed; in stress conditions, ABA actively down-regulates TOR to prioritize defense. At the molecular level, TOR kinase can directly phosphorylate ABA receptor proteins (PYL family proteins), preventing them from activating ABA signaling cascades (Fig. 1). This TOR-mediated PYL phosphorylation acts as a brake on ABA responses when growth is favorable, effectively raising the threshold for stress signaling activation and allowing growth to continue (Wang *et al*., 2018).

Conversely, ABA counteracts growth by engaging the SnRK1 kinase, a metabolic stress sensor that inhibits TOR. In Arabidopsi*s*, SnRK1 becomes active under energy stress and can repress TOR, shifting the cell toward catabolic and defense processes (Margalha *et al*., 2019). The ABA-activated SnRK2 kinases, which typically mediate the induction of stress-responsive genes, also have a hand in TOR modulation. In the absence of ABA, SnRK2 can form complexes with SnRK1 and keep it sequestered and inactive, allowing TOR to drive growth. When ABA levels rise during stress, this de-repression ends: SnRK1 is released and rapidly suppresses TOR activity, arresting growth and activating stress responses (Wang *et al*., 2018). ABA triggers a swift relocalization of SnRK1 within cells to shut down root meristem activity, allowing plants to rapidly pivot from growth to ‘defense mode’ via kinase signaling (Belda-Palazón *et al*., 2020). In summary, the TOR and ABA pathways are engaged in a constant tug-of-war: TOR signaling blocks ABA responses to favor growth when resources are plentiful, while ABA blocks TOR to favor survival under stress. This balanced antagonism ensures that growth is promoted only under safe conditions, and that stress responses dominate only when needed (Fig. 1).

TOR signaling intersects with hormone-mediated defense pathways beyond ABA. One clear example is TOR’s interaction with ethylene signaling. TOR phosphorylates the C-terminal domain of the central ethylene transducer ETHYLENE-INSENSITIVE 2 (EIN2), tethering it to the endoplasmic reticulum and preventing it from activating nuclear gene expression (Fu *et al*., 2021). EIN2 phosphorylation effectively suppresses ethylene-mediated defense programs when TOR is active, helping the plant prioritize growth. This same logic likely extends to other immune hormones such as salicylic acid (SA) and jasmonic acid (JA), whose signaling outputs are frequently amplified in TOR-inactive states (De Vleesschauwer *et al*., 2016; Song *et al*., 2017).

TOR’s role in the growth–defense trade-off is also modulated by the developmental context. In Arabidopsis, TOR inhibition has been found to confer minimal extra disease resistance in rapidly growing juvenile tissues, but stronger defense activation in mature leaves (Marash *et al*., 2024). This stage-dependent responsiveness suggests that developmental signals such as gibberellins during early growth might adjust the sensitivity of TOR signaling to immune cues.

From an evolutionary perspective, TOR’s role in balancing growth and defense in plants mirrors principles observed in other eukaryotes. In animals, mTORC1 promotes cell growth and proliferation while dampening autophagy and innate immune responses during nutrient-rich conditions, while AMP-activated kinase (AMPK) inhibits TOR activity under nutrient-poor conditions (Saxton and Sabatini, 2017). In plants, SnRK1 serves an analogous role, repressing TOR during stress and activating catabolic and defense-oriented programs, thereby shifting the cell toward catabolic, defense-oriented processes (Margalha *et al*., 2019). This conserved circuitry allows TOR and its interactors to integrate signals from nutrients, hormones, and stresses, and to allocate metabolic energy accordingly. The result is a dynamic and integrated management of cellular resources. Growth is promoted when conditions are favorable, but when challenges from pathogens or stress arise, TOR signaling is dialed down to activate defense programs and prioritize survival.

## Defense hormones as growth inhibitors

Classic defense hormones such as SA and JA are well-known to inhibit growth by antagonizing growth hormones [auxins, gibberellins (GAs), brassinosteroids] (Durbak *et al*., 2012). High SA stabilizes DELLA proteins (GA-pathway growth repressors) and suppresses auxin/GA-induced genes, causing growth inhibition (Rodriguez *et al*., 2016). JA triggers degradation of jasmonate ZIM-domain (JAZ) repressors, unleashing defense genes but also permitting DELLAs to accumulate (since JA dampens GA signaling), thereby blocking cell elongation (Yang *et al*., 2012). These hormone interactions create a push–pull between defense and growth: SA/JA activation often means growth pathways are shut down (De Vleesschauwer *et al*., 2018; Shields *et al*., 2022). However, examples are coming to light that show that some regulators have dual functions in both development and defense, blurring the line between these programs.

The stem cell regulator WUSCHEL (WUS), known for maintaining the shoot apical meristem, also has a role in immunity. WUS can directly activate defense programs in the meristem, binding and repressing certain methyltransferase genes, thereby inhibiting protein synthesis and impeding viral infection (He *et al*., 2022). In this way, a developmental gene is co-opted to safeguard the stem cell niche from pathogens. Similarly, the symbiotic fungi *Trichoderma* spp. provide a dual benefit to plants by simultaneously promoting growth and enhancing defense. These fungi produce auxin-like compounds and other growth stimulants while also priming plant immune responses (Hermosa *et al*., 2013). Finally, simultaneous disruption of both JAZ repressors and the light-sensing protein phytochrome B (PhyB) allows for robust growth and enhanced defense in some cases, challenging the traditional view of an obligatory trade-off between these processes (Campos *et al*., 2016). These examples underscore that the binary nature of growth–defense is not absolute; plants have evolved integrated mechanisms where developmental and immune signaling pathways converge.

Some of the most striking examples of growth–defense overlap are the severe developmental defects observed when plant immune pathways are constitutively active. Plant biologists have long noticed that mutants with heightened immune system activity (so-called ‘autoimmune’ mutants) are often small, developmentally stunted, and exhibit reduced fertility, even in the absence of pathogens (Johal, 2007; van Wersch *et al*., 2016; Mu *et al*., 2021). Recent research has uncovered some of the mechanistic underpinnings of these growth defects, revealing that it is not merely competition for resources and metabolic rebalancing, but also direct molecular crosstalk that makes vigorous growth and strong defense often mutually incompatible in plants.

**Fig. 2.**
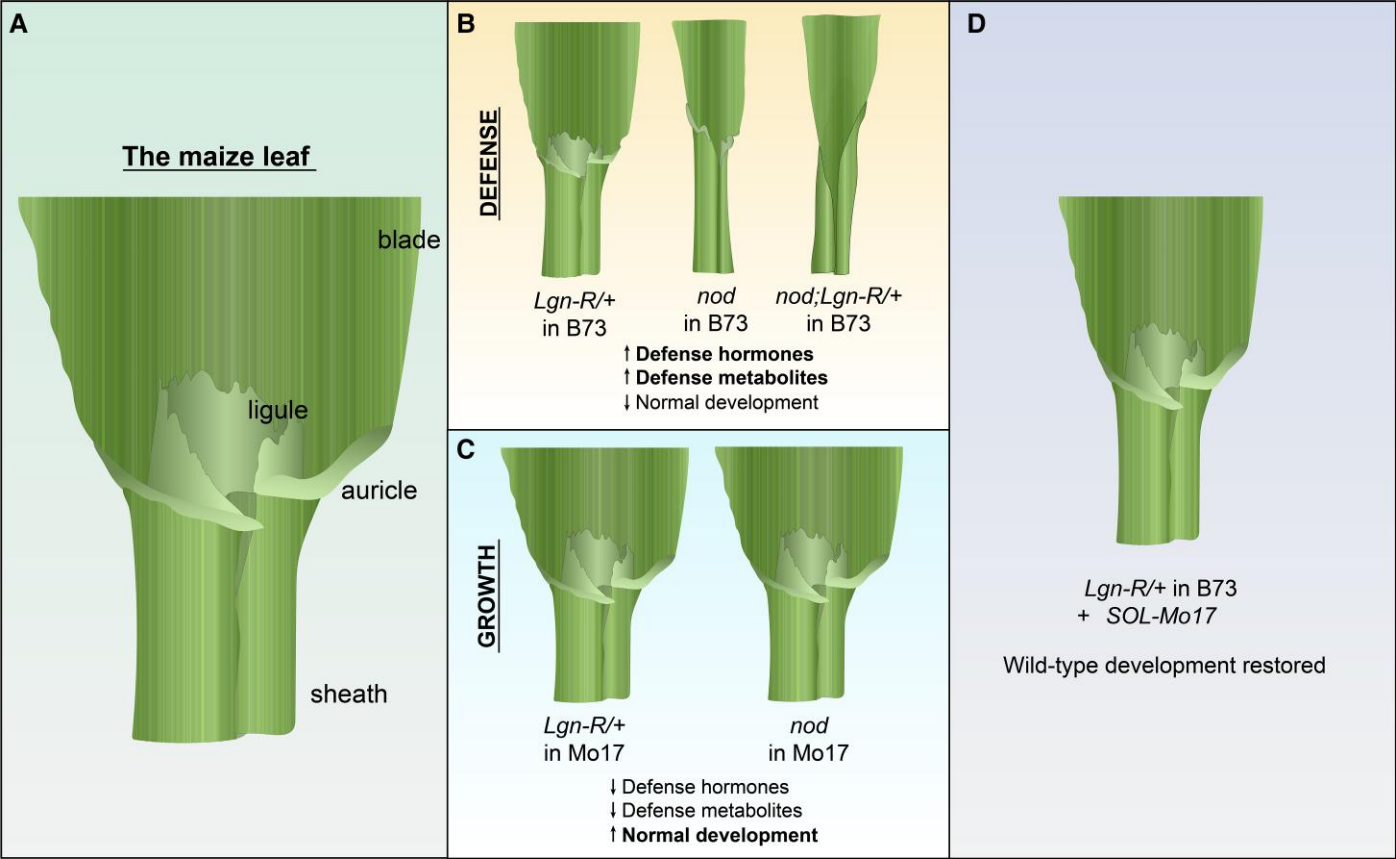
Maize leaf development is altered in response to changes in immune signaling. (A) The maize leaf is composed of four main parts: the sheath wraps around the stalk of the plant, the auricle is a hinge-like structure that is important for establishing the leaf angle, the ligule is a gasket-like structure that extends from the sheath along the stalk, and the blade is the major light-harvesting structure that extends away from the body of the plant. (B) The *Liguleless narrow* (*Lgn*) and *narrow odd dwarf* (*nod*) mutants in the B73 inbred line exhibit constitutive immune responses both metabolically and developmentally. They have noticeable defects in ligule and auricle formation that are exacerbated in the *nod Lgn-R/+* double-mutant. (C), These defects, however, are not apparent in the Mo17 background. (D) Introducing the Mo17 allele of *Sympathy for the ligule* (*Sol*) into the *Lgn-R* B73 background largely restores wild-type development and metabolism, preventing the stunted phenotype despite constitutive immune signaling.

A classical maize mutant with developmental defects and high immune activity is the *Liguleless narrow* (*Lgn*) mutant. First identified by Gerry Neuffer (University of Missouri, Columbia) during screening of mutants induced by ethyl methanesulfonate, *Lgn* has pleiotropic developmental defects, including the absence of a ligule. This gasket-like extension at the blade–sheath boundary has been the focus of some of the foremost maize developmental biologists in the last few decades (Fig. 2A) (Chaffey, 2000). It is a small, adaxial feature found on the leaves of most grass species whose expression is affected by, among others, the *liguleless* genes (Moreno *et al*., 1997; Richardson and Hake, 2022). Its sensitivity to developmental disturbances has made it a ‘canary in the coal mine’ for changes in fundamental growth and division processes such as cell division, organ patterning, and boundary formation. Importantly, *Lgn* mutants exhibit substantial reductions in organ size due to fewer and smaller cells, indicative of impaired cell proliferation and differentiation (Becraft *et al*., 1990; Fowler and Freeling, 1996; Rosa *et al*., 2017).

The *Lgn* gene encodes a plasma membrane-localized receptor-like kinase whose activity is necessary for many aspects of development beyond the formation of ligules. In some genetic backgrounds, such as the B73 inbred line, presence of the semi-dominant, kinase-dead mutant allele *Lgn-R* leads to defects including decreased height, decreased tassel branching, narrower leaves, and loss of the ligule at leaf margins (Fig. 2B) (Moon *et al*., 2013).

Another developmental mutant, *narrow odd dwarf* (*nod*), has pleiotropic, background-dependent developmental defects in many of the same traits as the *Lgn-R* mutant, and the protein is similarly localized to the plasma membrane. *nod* encodes a putative stretch-activated Ca^2+^-permeable channel orthologous to the Arabidopsis proteins MID1-COMPLEMENTING ACTIVITY 1 (MCA1) and MCA2 (Nakagawa *et al*., 2007; Furuichi *et al*., 2012; Rosa *et al*., 2017; Yoshimura *et al*., 2021). In B73, the *nod* mutant is severely dwarfed, has narrow leaves, increased tillering, and complete loss of the ligule and auricle (Fig. 2B).

Despite their similar developmental defects, we recently found that these genes have an additive effect on mutant phenotypes rather than interacting epistatically (Abraham-Juárez *et al*., 2022). Protein–protein interaction experiments have revealed that they can interact, and that LGN is able to phosphorylate NOD *in vitro*. Loss of both *nod* and *Lgn* together has an additive effect on the transcriptional, metabolic, and phenotypic defects of either mutant. *nod Lgn-R/+* double-mutants have extremely narrow leaves, complete loss of the ligule and auricle, heavy tillering, with the main shoot often failing entirely (Fig. 2B).

The effects of the *NOD* and *LGN* genes on development do not appear to be direct. Rather, they may be responsible for transducing important immune signals. Both *nod* and *Lgn-R/+* single-mutants induce expression of genes involved in pathogen defense and increase production of secondary metabolites (Moon *et al*., 2013; Dixon and Dickinson, 2024). These transcriptional and metabolic signals are exacerbated in the *nod Lgn-R/+* double-mutant, where immune signaling is constitutively induced and growth is severely impaired (Abraham-Juárez *et al*., 2022). The double-mutant indicates that NOD and LGN have overlapping impacts on the expression of development- and immunity-related genes. In the absence of these immune signals, wild-type-like development cannot proceed.

The *nod* and *Lgn-R/+* mutants are good examples of the trade-off between growth and stress responses and, more specifically, show how these processes can be deeply intertwined rather than necessarily distinct and alternating. These observations align with a model where disrupted growth processes (potentially linked to altered TOR activity or downstream targets) lead to a compensatory increase in defense gene expression in a dynamic response to compromised developmental conditions.

Curiously, compared to B73, the severity of these phenotypes is significantly reduced in the A619 and Mo17 inbred backgrounds. In Mo17 in particular, both single-mutants are nearly indistinguishable from the wild type, especially in the case of *Lgn-R*. (Figure 2C) (Rosa *et al*., 2017; Abraham-Juárez *et al*., 2022). Instead, inbred-specific modifiers appear to cause variability in penetrance. For example, when the Mo17 version of the *Sympathy for the ligule* (*Sol*) gene is crossed into the *Lgn-R/+* mutant in B73, it largely eliminates the developmental defects (Fig. 2D) (Anderson *et al*., 2019). These results suggest that a heightened immune response does not always irreversibly alter development. Valuable genetic modifiers such as the Mo17 version of *Sol* can rescue growth and development even under constant defense activation. The ability of unique modifiers to critically regulate development and immunity demonstrates the complexity of the growth–defense relationship across the plant kingdom. Yet despite emerging exceptions, many molecular studies continue to emphasize the antagonistic relationship between growth and defense, particularly through the lens of hormone signaling.

## Jasmonate signaling in growth and defense

A major reason why plant immunity dampens growth is the action of defense hormones. These molecules, such as SA and JA, antagonize growth-promoting hormones. When a plant detects a pathogen, SA and/or JA levels surge (SA mainly for biotrophic pathogens, JA for herbivores/necrotrophs) and trigger transcriptional reprogramming towards defense. High SA, for example, induces pathogenesis-related genes and strengthens defenses, but it simultaneously can suppress growth pathways, partly by interfering with hormones such as auxin, GA, and brassinosteroid. TOR signaling, which promotes growth, has been shown to antagonize SA and JA signaling; when SA/JA signaling is strongly activated, TOR is often down-regulated (De Vleesschauwer *et al*., 2018; Shields *et al*., 2022).

But the roles these hormones play are not as clear-cut as they once seemed, not only with respect to their complex ‘crosstalk’ to regulate growth and metabolism, but additionally in their individual roles as signaling molecules. Along with antagonizing growth-promoting hormones, SA also stabilizes or activates growth-repressing factors. For instance, SA can promote the accumulation of DELLA proteins (growth inhibitors in the GA pathway) and repress genes induced by auxin or GA, thereby blocking cell expansion and division. Maize mutants with constitutively high SA show dwarf phenotypes with elevated DELLA activity and reduced expression of growth-related genes (Rodriguez *et al*., 2016). JA, on the other hand, triggers the degradation of JAZ repressors. JAZ degradation unleashes defense responses but can also reduce growth by crosstalk with the GA system, since JA and GA typically have opposing effects on DELLA. JA signaling tends to allow DELLA to accumulate (since GA signaling is often attenuated during defense), leading to inhibited cell elongation.

Beyond its role in defense, however, JA is critical for many steps of maize development. Some mutants in the JA biosynthesis pathway fail to produce staminate flowers (Subramaniam and Bartlett, 2023), since the molecule is required to prevent silk formation in the tassel (DeLong *et al*., 1993; Acosta *et al*., 2009; Best and Dilkes, 2023). Altered development is also seen in *Tasselseed5* (*Ts5*), a dominant mutant in which the maize homolog of Arabidopsis CYP94B1, ZmCYP94B1, is mis-expressed. This leads to accumulation of certain JA-related intermediates, misexpression of other JA signaling genes, and altered sex determination. More specifically, ZmCYP94B1 normally functions to hydrolyse bioactive jasmonate, jasmonoyl-isoleucine (JA-Ile), into oxidized JA, a necessary step for pistil abortion. Furthermore, while its developmental effects have been well-described, ZmCYP4B1 has clear roles in response to wounding as well. The *Ts5* mutant shows dramatic differences in levels of wound-induced jasmonates when compared with wild type plants, both before and after wounding (Lunde *et al*., 2019). Jasmonates are just one example of a secondary metabolite with roles in these opposing programs. This study is a clear demonstration of the complexities of hormone signaling and metabolism as related to growth and defense, and further dismantles the idea of strict opposition between growth and stress physiology.

## Maize-specific innovations in JA signaling

Maize provides even more examples of species-specific mechanisms that can partially uncouple growth from defense. A study in 2024 discovered an ‘unexpected protein connection’ involving the JA pathway. Maize has multiple *coi1* genes (*COI1* encodes the F-box receptor for JA) (Feiz *et al*., 2024) and, surprisingly, maize COI1 proteins not only target JAZ repressors (as in typical JA signaling), but also target DELLA growth repressors for degradation. In Arabidopsis and rice, COI1’s main role is to bind JA and trigger JAZ degradation, unleashing defense genes (Hou *et al*., 2010). In the absence of JA, JAZ proteins sequester DELLAs, such that JA acts as a growth inhibitor in those plants. When COI1 is mutated in Arabidopsis, JA signaling is switched off and plants grow taller since DELLAs get stuck bound to JAZ and are not able to inhibit growth genes (Feiz *et al*., 2024). In maize, however, knocking out all four *coi1* genes causes reduced rather than enhanced growth. Maize COI1s appear to directly facilitate the removal of DELLAs when JA is present. So, when JA levels rise (e.g. under pest attack), maize can keep growing because its COI1 simultaneously activates defense (via JAZ degradation) and alleviates growth suppression (via DELLA degradation). Importantly, this buffering mechanism does not eliminate the growth penalties of severe immune activation; it simply raises the threshold at which defense hormones begin to reduce growth in maize. Essentially, the JA pathway in maize might contain a built-in mechanism to buffer its anti-growth effect.

As LGN and NOD both transduce signals that feed into stress and defense programs, their constitutive mis-activation offers a direct test of maize’s JA/SA buffering capacity. Wild type plants usually balance growth and immunity, consistent with the ability to tolerate moderate defense activation. By contrast, the constitutive immune signaling in *Lgn* and *nod* mutants overwhelms even maize’s buffering mechanisms (except in the Mo17 background), and results in the classic dwarf-and-defense phenotype exacerbated in the double-mutant. This maize exception is telling—when immune signaling is forced on by mutations in *Lgn* or *nod*, even maize’s built-in buffer may be overwhelmed.

Clearly, maize is not immune to trade-offs: severe defense activation (such as by systemic diseases or heavy infestations) will reduce yield, as in any crop. But the maize examples highlight species- (or potentially family-)specific innovations that mitigate the growth–defense antagonism. Thus, further investigation of growth–defense balancing in maize, especially in response to JA/SA signals, could provide important insights for decoupling this trade-off. Since TOR is such a highly conserved growth regulator (maize TOR genes can complement Arabidopsis *tor* mutants, suggesting functional conservation), studying how maize links its unique metabolic balancing system to TOR signaling could lay the groundwork for transferrable approaches to de-coupling this trade-off in other crops. In our own work, we have begun to map the maize TOR signaling network under TOR-activating and -inactivating conditions, using functional genomic approaches to pinpoint both conserved and maize-specific TOR effectors. We expect that these data will reveal the precise regulatory nodes through which TOR regulates development in response to nutrient availability, laying a mechanistic foundation for targeted interventions that could tailor growth in not only maize but across diverse crop species.

## Light signals and shade avoidance

Effective light harvesting is critical for photosynthesis, phytohormone signaling, maintenance of circadian rhythms, and growth and development more broadly, but environmental cues from light also profoundly influence the growth–defense balance. As such, plants have evolved incredibly sensitive mechanisms for sensing both the quality (wavelengths) and quantity of light. Land plants do this through a number of photoreceptors, including by monitoring the ratio of red (R) to far-red (FR) light with phytochromes (Quail, 2002; Chen *et al*., 2004).

Red light is more photosynthetically stimulating than far-red light but can be captured by the vegetative canopy of neighboring plants. Far-red light, instead, is less stimulating and passes through or is reflected by nearby greenery. By monitoring the ratio of R to FR light, plants can determine if they are in bright, direct light optimal for photosynthesis or in the shade, and adjust their growth accordingly (Smith, 1982; Shi *et al*., 2019; Tan *et al*., 2022). In maize, as in the model plant Arabidopsis, this process is primarily coordinated by PhyB (Franklin and Quail, 2009). Maize encodes two paralogues of *phyB*, which are largely functionally redundant (Sheehan *et al*., 2007; Busche *et al*., 2023).

Prolonged exposure to low R:FR leads to dramatic changes in growth habit, including stem elongation, a decrease in chlorophyll production, and stunted leaf development that are collectively known as the shade avoidance syndrome (SAS), reminiscent of skotomorphogenic growth in etiolated seedlings (Smith and Whitelam, 1997; Kebrom and Brutnell, 2007). Shade-induced auxin is a key mediator of elongation. Elevated auxin levels under low R:FR conditions have been shown to activate TOR signaling, thereby promoting the protein synthesis and cell proliferation needed for stem elongation (Pfeiffer *et al*., 2016). This light–auxin–TOR axis links environmental light cues to the growth machinery, integrating shade avoidance, through PhyB signaling, into the plant’s overall energy and growth regulation network.

Under canopy shade, when PhyB is inactive, plants prioritize vertical growth at the expense of immune preparedness. Low R:FR conditions or loss of PhyB function attenuate JA signaling and reduce resistance to foliar pathogens such as *Botrytis cinerea* (Cerrudo *et al*., 2012). This is because PhyB inactivation stabilizes PHYTOCHROME-INTERACTING FACTOR (PIF) transcription factors that promote elongation growth while simultaneously repressing many defense-related genes. The result is a plant that grows taller in shade but becomes more susceptible to pests and diseases. By contrast, in high-light environments (high R:FR), active PhyB constrains excessive elongation and allows robust defense responses. Indeed, active PhyB has been shown to enhance JA-mediated defenses, by promoting JA biosynthesis and signaling upon pathogen attack (Xiang *et al*., 2021). While multiple phytochromes (PhyA, PhyB, etc.) mediate shade responses, canopy shade avoidance is predominantly governed by PhyB activity.

Thus, PhyB serves as a molecular switch that determines whether to invest in so-called ‘normal’ growth or to shift into the shade-avoidance developmental pattern as an adaptive strategy, resulting in the characteristic SAS phenotypes (Smith and Whitelam, 1997). More recent work has shown that light-derived signals are integrated by the TOR pathway, which in turn not only modulates hormone networks but also influences alternative splicing outcomes to fine-tune development (Pfeiffer *et al*., 2016; Riegler *et al*., 2021). Although modern maize has been bred to attenuate SAS, these molecular insights underscore how photoreceptors such as PhyB directly link external light cues to internal transcriptional and hormonal programs that impact development and balance growth and defense. Notably, a study on tomato has shown that FR enrichment (simulating canopy shade) dampens JA-mediated defenses and increases susceptibility to *B. cinerea* (Courbier *et al*., 2020). Under low R:FR, PhyB-inactivated tomato plants were found to have elevated sugar levels and reduced immune responses, leading to larger pathogen lesions. This work demonstrates that shade-induced growth can indeed compromise defense in crop species.

## Growth at the cost of defense

So far, we have discussed how stress can affect development, as well as the overlapping roles certain genes play in development and immune signaling. We have examined the trade-off between growth and defense when growth is suboptimal, but this dialectical relationship also persists in situations in which growth is enhanced.

When growth is promoted, plants repress expression of defense-related genes, often leading to increased susceptibility to pathogens and more serious impacts from abiotic stress. This trade-off occurs even when growth is promoted by a transgene. The human RNA demethylase FAT MASS AND OBESITY ASSOCIATED PROTEIN (FTO) has been found to positively regulate growth in rice, potatoes, and Arabidopsis (Yu *et al*., 2021; Markel *et al*., 2024). Expression of this transgene leads to increases yield and yield-associated traits including floral and fruit number, and mass. In all three species, RNA-seq experiments showed that genes associated with ‘defense response’ and ‘regulation of defense response’ were significantly repressed in mutants expressing *FTO*. While no pathology studies have been performed on *FTO* transgenics to date, increased susceptibility to diverse stresses has been repeatedly demonstrated in plants with increased growth (Zavala *et al*., 2004; Huot *et al*., 2014). These data suggest that although the switch between growth and defense can be regulated genetically, it is possible that growth *per se* can lead to repression of defense-related metabolism and gene expression rather than through precise genetic or metabolic regulation alone.

Consequently, it may not be surprising that attempts to overexpress *TOR* to promote growth have produced mixed results (Deprost *et al*., 2007; Bakshi *et al*., 2017). A study in rice showed that overexpression (OX) of *OsTOR* was sufficient to increase yield and yield-related phenotypes including longer shoots and increased tillering (De Vleesschauwer *et al*., 2018). The *OsTOR*-OX lines displayed increased transcription of genes involved in ribosome biogenesis and cell division, characteristic outputs of increased TOR activity. They also significantly repressed transcripts related to abiotic stress response and pathogen defense, specifically genes involved in the metabolism of JA, ethylene, ABA, auxins and brassinosteroid. In fact, the *OsTOR*-OX plants were more susceptible to the pathogen *Xanthomonas oryzae* pv*. oryzae*, confirming that growth induced by overexpression of *TOR* has negative impacts on defense. These negative consequences drive home the importance of continued fundamental studies of TOR signaling, since a nuanced understanding of the regulatory network is required to decouple its positive effects on growth from its negative effects on stress susceptibility.

## Conclusions

Growth is complex, and most research in maize has been focused on understanding the aspects that ultimately maximize yield. The scale, structure, and timing of shoot development heavily contribute to this complex agronomic trait, complicated further by environmental effects and genotype-by-environment interactions. Maize breeders have performed important work to optimize and prioritize growth across diverse environments to maximize yield, but this has come at the cost of decreased sensitivity to abiotic and biotic stresses. The ‘choice’ that plants make between growing and defending from stresses happens on many scales: upon acute exposure, immediate cellular and subcellular changes occur, while chronic stress exposure stimulates long-term developmental changes. The switch from growth to defense, however, is not well-defined and differs across contexts and species.

Discovering more about both the short- and long-term strategies plants use to address stress is increasingly important as environmental stress becomes more impactful on agriculture globally. The Intergovernmental Panel on Climate Change recently reported that average global surface temperature has increased by 1.1 °C since the industrial revolution (IPCC, 2023). The damage from this temperature increase has been manifold; the Secretary-General of the United Nations António Guterres has called the impacts of climate change ‘an atlas of human suffering and a damning indictment of failed climate leadership’. But plant scientists are working on solutions (Eckardt *et al*., 2023). Improvements to our farming systems and the plants we grow in them offer hope for mitigating our impacts on the climate and reducing threats to our food systems.

As we face more extreme and unpredictable climates, we must re-examine the targets and strategies we use for crop improvement. Plant biologists tend to emphasize the immediate impacts of stress on physiology, such as reduced photosynthetic efficiency or induced synthesis of protective secondary metabolites, and the medium-term impacts of these physiological changes on overall growth rates, i.e. various ‘growth–defense trade-offs’. But there is growing appreciation that plant stress also causes lasting changes to plant developmental patterning beyond simple growth rates, impacting leaf shape, shoot architecture, and reproductive strategy, which go on to critically impact yield.

**Fig. 3.**
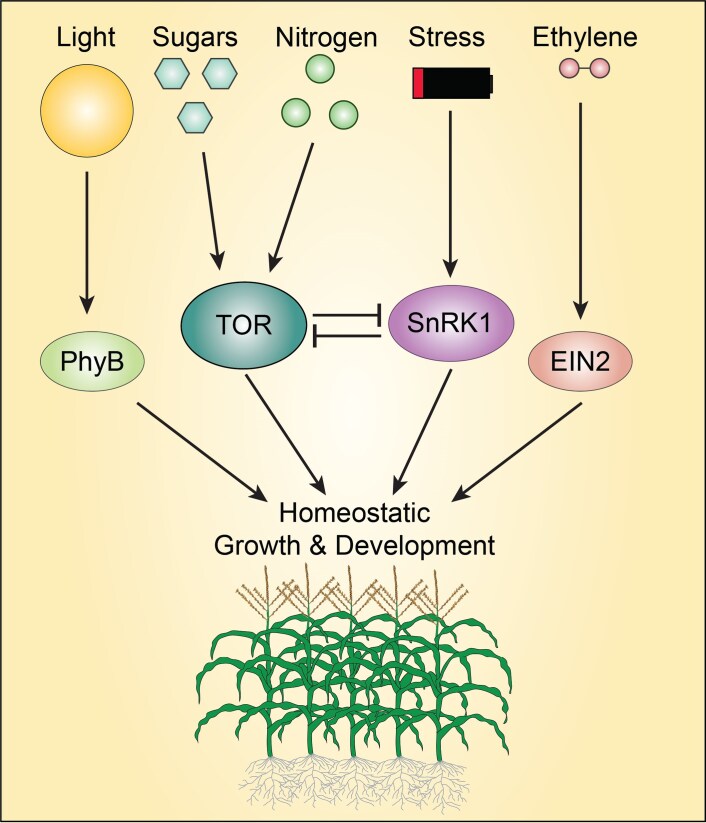
Diverse signaling pathways converge to regulate growth and development. Signals from an array of regulators and downstream effectors must be effectively integrated to maintain homeostatic growth as internal physiology and external environments continuously change. Many of these processes exhibit complex crosstalk with one another. Inputs such as light, sugar, and nitrogen promote TOR activity, which stimulates growth and development. In contrast, stress activates SnRK1, which antagonizes TOR to conserve resources and initiate defense responses. PhyB senses light quality and regulates TOR-linked developmental cues such as shade avoidance, while EIN2 mediates ethylene responses that influence both growth and immunity. Understanding how these pathways converge provides a foundation for engineering crops that can sustain yield while withstanding increasingly unpredictable stresses. EIN2, ETHYLENE-INSENSITIVE 2; PhyB, phytochrome B; SnRK1, SNF1-RELATED KINASE; TOR, TARGET OF RAPAMYCIN.

A sustainable agricultural system will require resilient crop plants that can withstand periods of stress without rewiring developmental patterns in ways that have long-term detrimental effects on yield, especially in the face of transient stress (Lutt and Brunkard, 2022; Zurek *et al*., 2022). A prime example of this from the history of domestication is the shade avoidance syndrome: maize breeders were successful in largely diminishing the SAS through traditional breeding methods and obtained better yields without compromising plant vitality. Modern technologies will help expedite this process, but only when plant scientists and maize breeders have a clearer understanding of the physiology and developmental rewiring that occurs when plants encounter stress (Fig. 3). To this end, we advocate for a deeper and more mechanistic understanding of how the TOR–SnRK1 signaling networks coordinate energy and stress signals with plant growth and development in the world’s most important crops.

## Abbreviations

ABA: Abscisic acid
AMPK: AMP-ACTIVATED KINASE
COI1: CORONATINE-INSENSITIVE1
eIF2α: EUKARYOTIC INITIATION FACTOR 2 alpha
FTO: FAT MASS AND OBESITY ASSOCIATED PROTEIN
GCN2: GENERAL CONTROL OF NONDEREPRESIBLE 2
GA: gibberellin
JA: jasmonic acid
JAZ: jasmonate ZIM-domain repressor proteins
LGN: LIGULELESS NARROW
LST8: LETHAL WITH SEC THIRTEEN 8
MCA1 and 2: MID1-complementing activity 1 and 2
NOD: NARROW ODD DWARF
OX: overexpression
PAR: photosynthetically active radiation
PP2A: PROTEIN PHOSPHATASE 2A
RAPTOR: REGULATORY ASSOCIATED PROTEIN OF mTOR
SA: salicylic acid
SAS: shade avoidance syndrome
SnRK1: SNF1-RELATED KINASE
SOL: SYMPATHY FOR THE LIGULE
TAP46: TYPE 2A PHOSPHATASE-ASSOCIATED PROTEIN OF 46 kDa
TOR: TARGET OF RAPAMYCIN
